# CO_2_ driven endotracheal tube cuff control in critically ill patients: A randomized controlled study

**DOI:** 10.1371/journal.pone.0175476

**Published:** 2017-05-11

**Authors:** Gennaro De Pascale, Mariano Alberto Pennisi, Maria Sole Vallecoccia, Giuseppe Bello, Riccardo Maviglia, Luca Montini, Valentina Di Gravio, Salvatore Lucio Cutuli, Giorgio Conti, Massimo Antonelli

**Affiliations:** Department of Anesthesiology and Intensive Care, Sacro Cuore Catholic University, A. Gemelli Hospital, Rome, Italy; Postgraduate Institute of Medical Education and Research, INDIA

## Abstract

**Background:**

To determine the safety and clinical efficacy of an innovative integrated airway system (AnapnoGuard^™^ 100 system) that continuously monitors and controls the cuff pressure (P_cuff_), while facilitating the aspiration of subglottic secretions (SS).

**Methods:**

This was a prospective, single centre, open-label, randomized, controlled feasibility and safety trial. The primary endpoint of the study was the rate of device related adverse events (AE) and serious AE (SAE) as a result of using AnapnoGuard (AG) 100 during mechanical ventilation. Secondary endpoints were: (1) mechanical complications rate (2) ICU staff satisfaction; (3) VAP occurrence; (4) length of mechanical ventilation; (5) length of Intensive Care Unit stay and mortality; (6) volume of evacuated subglottic secretions.

Sixty patients were randomized to be intubated with the AG endotracheal-tube (ETT) and connected to the AG 100 system allowing P_cuff_ adjustment and SS aspiration; or with an ETT combined with SS drainage and P_cuff_ controlled manually.

**Results:**

No difference in adverse events rate was identified between the groups. The use of AG system was associated with a significantly higher incidence of P_cuff_ determinations in the safety range (97.3% vs. 71%; *p*<0.01) and a trend to a greater volume of aspirated SS secretions: (192.0[64–413] ml vs. 150[50–200], *p* = 0.19 (total)); (57.8[20–88.7] ml vs. 50[18.7–62] ml, *p* = 0.11 (daily)). No inter-group difference was detected using AG system vs. controls in terms of post-extubation throat pain level (0 [0–2] vs. 0 [0–3]; *p* = 0.7), hoarseness (42.9% vs. 75%; p = 0.55) and tracheal mucosa oedema (16.7% vs. 10%; *p* = 0.65).

Patients enrolled in the AG group had a trend to reduced VAP risk of ventilator-associated pneumonia(VAP) (14.8% vs. 40%; *p* = 0.06), which were more frequently monomicrobial (25% vs. 70%; *p* = 0.03).

No statistically significant difference was observed in duration of mechanical ventilation, ICU stay, and mortality.

**Conclusions:**

The use AG 100 system and AG tube in critically ill intubated patients is safe and effective in P_cuff_ control and SS drainage. Its protective role against VAP needs to be confirmed in a larger randomized trial.

**Trial registration:**

ClinicalTrials.gov NCT01550978. Date of registration: February 21, 2012.

## Background

Maintaining the endotracheal tube (ETT) cuff appropriately inflated plays a crucial role in the management of intubated patients[1–5]. Overinflation of the ETT cuff may compromise capillary perfusion causing ischemic tracheal wall damage, ulcerations and tracheal stenosis[6, 7]. Conversely, suboptimal tracheal sealing, usually associated with P_cuff_ values below 20 cmH20, may result in significant fluid leakage around the cuff, which is a crucial risk factor for ventilator associated pneumonia (VAP)[8, 9]. However, up to now, a ‘magic number’ for the optimal sealing of the trachea is not available yet. During mechanical ventilation, secretions contaminated with oropharyngeal pathogens pool in the subglottic region (tracheal region between the ETT cuff and the vocal cords) and enter the lower airways via microaspiration. Subglottic secretions drainage (SSD), obtained with specially designed ETT with a intra-mural lumen, has been observed to significantly reduce the incidence of VAP[10]. Drainage of sub-glottic secretions may be performed intermittently or continuously, with varying efficacy in clearing the subglottic space, while often causing tracheal mucosa lesions secondary to the applied suction[11]. AnapnoGuard^™^ (AG) 100 System is a new device for the management of intubated patients. The AnapnoGuard system provides continuous P_cuff_ control, detecting air leakage from the lungs by measuring the carbon dioxide (CO_2_) level in the subglottic space. Concomitantly, the system evacuates secretions from above the sub-glottic space by simultaneously rinsing the space with saline from one port of the tube while performing suction from the two other suction ports. Both a more efficient SSD and a more accurate P_cuff_ control may reduce the frequency and the amount of micro-inhalation episodes which are the ‘primum movens’ for the development of lower respiratory tract infections. Indeed, the clinical implementation of such innovative device is expected to minimize the clinical complications associated with over-inflation of ETT cuff along with the reduction of ventilator-associated infectious complications burden. This innovative technology has received both Food and Drug Administration (FDA) and Conformité Européenne (CE) approval[http://www.hospitech.co.il] and it has been shown to be feasible and safe in preclinical and preliminary clinical investigations[12–14].

We designed this prospective randomized controlled study to assess the safety and clinical efficacy of AG 100 system in critically ill, mechanically ventilated patients, as compared to ventilator care including manual P_cuff_ control and evacuation of subglottic secretions.

## Methods

### Study design

This open-label, double-arm, randomized controlled trial was conducted in the 18-bed general ICU of a 1,500 bed tertiary teaching hospital in Rome, from February 2012 to April 2015. According to Italian law, the protocol was approved by Catholic University’s Ethical Committee (P/137/CE/2012) and written informed consent was obtained from the patient or the legally authorized representative. The trial was registered on www.clinicaltrials.gov (NCT01550978).

### Study population and randomization

ICU admitted patients, older than 21 years, were eligible for enrolment if they required tracheal intubation (7.5 mm or 8.0 mm diameter tube) and were expected to need mechanical ventilation for 12 hours or longer. The other inclusion criteria to be fulfilled were: (1) absence of clear signs of pneumonia and lung contusion on chest x- ray; (2) connection of the ETT to the SS aspiration system less than 6 hours from intubation; (3) no fever or fever from non-lung origin. Patients were excluded if they (1) had been treated with mechanical ventilation during the last three months, (2) had facial, oropharyngeal or neck trauma, (3) were morbidly obese (Body Mass Index > 40 Kg/m^2^), (4) were pregnant, (5) were expected to be ventilated in prone position, (6) had been difficult to intubate (more than three attempts). Withdrawal criteria were: (1) refusal of the subject/legal representative to continue the trial; (2) any significant adverse event that, upon the investigator opinion, could be related to the AnapnoGuard system; (3) significant protocol deviations; (4) less than 12 hours connection to SS aspiration system; (5) any chest x-ray pathological sign in the 12 hours following intubation; (6) reintubation. Patients were assigned in a 1:1 ratio to treatment groups according to a randomization list generated by validated software using block randomization with fixed block length of 10. Investigators were blinded to block length; microbiology and laboratory personnel were blinded to group assignments. Patients randomized in the study group (AG) were intubated with AnapnoGuard ETT (ID 8.0 mm polyvinylchoride [PVC] tube with ellipsoidal shape, thin wall polyurethane (PU) cuff with dual suction lines and an extra venting line), and connected within 6 hours to the AnapnoGuard 100 control unit (Hospitech Respiration LTD., Petach-Tikva, Israel; ID 8 mm, OD 11.9) which provided continuous P_cuff_ regulation and evacuation of SS from above the cuff. The AnapnoGuard 100 device controls the cuff pressure in two layers and the user is asked to set Upper and Lower pressure limits. The optimal Target pressure is set by the system within the preset limits. The 1st control layer is similar to the standard cuff pressure controllers, where the system constantly keeps the target pressure constant. The 2nd control layer is the one where the device automatically adjusts the target pressure based on the CO_2_ levels/leaks above the cuff. The AnapnoGuard 100 device incorporates a high sensitivity capnograph at low levels of CO_2_. The AnapnoGuard ETT includes one extra lumen in compare to common suction ET tubes. This extra lumen (Vent/CO_2_) is used for venting, rinsing and also for air sampling from the subglottic space to measure CO_2_ levels above the cuff. The ETT is connected to the device with a three lines harness, where one line allows air sampling from the subglottic space to the capnograph. The device operates automatically in cycles, where every few min, air sample (suction) from above the cuff is taken to measure CO2 levels above the cuff in order to assess the level of the leak. CO_2_ level thresholds which correlate to leaks were set through extensive animal studies. If measured CO2 levels above the cuff is below the threshold, the device reduces the target pressure by 1 mmHg. If the CO2 level is above the threshold, the system increases the target pressure by a formula. All within the pressure limits set by the use. In case the system reaches the upper pressure limit and a leak is still detected, then visual and audio alert is activated

Patients randomized in the control group (CG) were intubated with the TaperGuard Evac ETT (Mallinckrodt Medical, Athlone, Ireland; ID 7.5–8.0 mm), incorporating a dorsal additional lumen ending above the PVC conic cuff, and connected within 6 hours to the system for SS continuous aspiration (Continuous Aspiration of Subglottic Secretions [CASS] regulator, Boehringer Laboratories, Inc.). In both groups, SS drainage and P_cuff_ management were continued until the end of mechanical ventilation or death. Predefined target P_cuff_ was 24–40 cm H_2_O. In the AG group, P_cuff_ control was obtained using AG 100 system. In the control group, routine care of the tracheal cuff was performed using a manual manometer (Ambu Cuff Pressure Gauge; Ambu A/S, Ballerup, Denmark) to check and adjust P_cuff_ at least three times a day (once per nurse shift).

In the study group, secretions were evacuated by the AnapnoGuard 100 system, utilizing intermittent simultaneous rinsing and suction. In the control group, secretions were evacuated using the Ohio suction device, preset to the intermittent mode. In both cases, the volume of secretions accumulated in the secretion canister were manually measured on a daily basis and documented in the CRF. In the study group, where irrigation was performed by the AnapnoGuard 100 system, the volume of the irrigated saline was deducted from the total volume collected in the secretion canister, to present the net volume of secretions.

### Endpoints

The primary endpoint of the study was the rate of device related adverse events (AE) and serious AE (SAE) as a result of using AnapnoGuard 100 during mechanical ventilation. Definitions of AE and SAE are explained in the Supporting information (S1 File). Secondary endpoints were: (1) mechanical complications rate due to ETT over inflation evaluated during the 48 hours post-extubation (S1 File); (2) ICU staff satisfaction (S1 File); (3) VAP occurrence; (4) length of mechanical ventilation (MV); (5) length of Intensive Care Unit stay (ICU) and mortality; (6) volume of evacuated subglottic secretions.

### Data collection and definitions

A research nurse and senior physicians daily collected data which were entered into a dedicated case-report form and documented in the ICU’s electronic chart (Digistat) (S1 File). The simplified acute physiology score II (SAPS II) and sequential organ failure assessment (SOFA) score were used to assess severity [15, 16]. In both groups, identical measures for the prevention of VAP were applied. Clinical suspicion of VAP was established according to current recommendations [17] (S1 File). Tracheal mucosal alteration and lesions were evaluated by bronchoscopic inspection (S1 File). Septic shock was defined as recommended by the American College of Chest Physicians/ Society of Critical Care Medicine Consensus Conference Committee[18]. Empirical and definite antimicrobial therapies were prescribed according to local protocols.

### Sample size calculation and statistical analysis

Given previous preliminary data reporting no AE/SAE while using AG 100, we considered 80% of study patients with no AE/SAE to be an appropriate clinical target for AnapnoGuard 100 success rate. Assuming the same success rate in the control group, we calculated that 50 patients (25 per each group) were needed to show that AnapnoGuard 100 success (no AE or SAE the during intubation period) is at least 80%. This is based on a power analysis using the normal approximation for the binomial, with one-sided α = 0.05. To account for an approximate 15% dropout rate, we set a total number of 60 patients (S1 File).

## Results

### Patients characteristics

**Fig 1:**
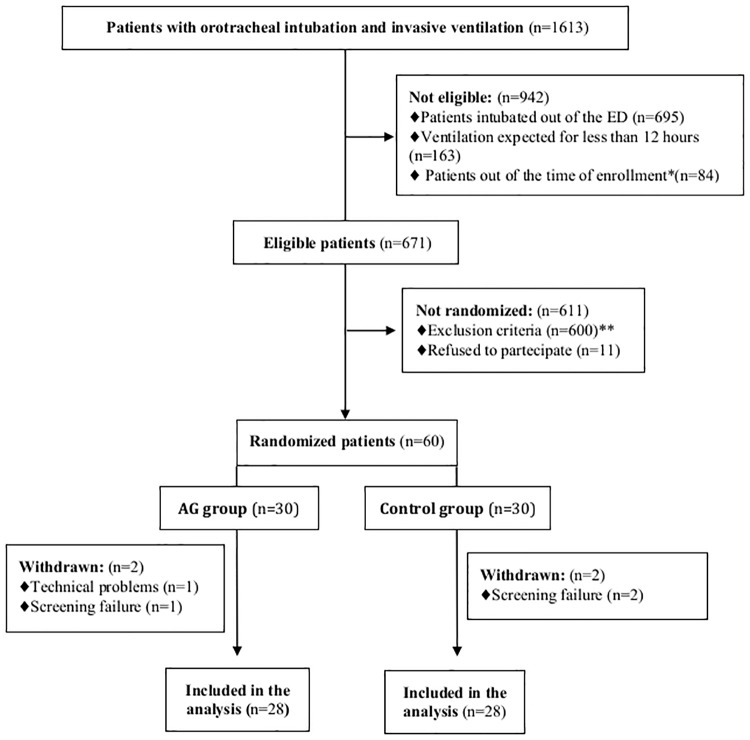
Flow chart of study inclusion process. *ED*, Emergency Department; *BMI*, Body Mass Index *Patients connected to either AG 110 system or subglottic suction system after 6 hours from tracheal intubation **The reasons for excluding patients at admission were: suspicion of pneumonia and lung contusion on chest X ray (n = 375); mechanical ventilation during previous three months (n = 58); facial, oropharingeal and neck trauma (n = 123); difficult intubation (n = 28); BMI >40 (n = 16).

During the study period, out of 1613 intubated mechanically ventilated patients, 671 were eligible for the inclusion, 60 enrolled in the study, 56 were included in the analysis and no one was lost at the follow-up (Fig 1). Main reasons for exclusion were the suspicion of pneumonia or lung contusion on chest X ray (62.5%) and the presence of facial/neck trauma (20.5%).

Table 1 describes the characteristics of the patients at inclusion. The two groups (AG and controls) were similar in terms of demographics and clinical presentation. Illness severity was also comparable: SAPS II score (55.32 vs. 52.17, *p* = 0.5), SOFA score (5 [4–6] vs. 5[4–6], *p* = 0.6) and incidence of septic shock at intubation (32.1% vs. 11%, *p* = 0.1) Main comorbidities were diabetes (28.5%) and cardiovascular diseases (16.1%). The leading three reasons for admission and mechanical ventilation were: neurological disease (32.1%), sepsis (21.4%), and respiratory failure (21.4%). The majority of the patients were ventilated using pressure support (58.9%) and no differences were detected regarding either peak inspiratory pressure (*p* = 0.22) or positive end-expiratory pressure (*p* = 0.22). Mean respiratory rate was significantly higher in the AG group (18.9 vs. 16.5, *p* = 0.03).

**Table 1:** Baseline patient characteristics.

	AG 100 Group (*n* = 28)	Control Group (*n* = 28)	*P* value
**Demographics, comorbidities and presenting features**			
Age, years	67.5±17.4	65.8±11.3	0.4
Male sex, N (%)	21 (75%)	21 (75%)	1
Diabetes mellitus, N (%)	10 (35%)	6 (21.4%)	0.38
Renal failure, N (%)	2 (7.1%)	1 (3.5%)	1
Cardiovascular diseases, N (%)	4 (14.3%)	5 (17.9%)	1
COPD, N (%)	0	1 (3.6%)	0.9
Immunosuppressive status, N (%)	0	0	-
SAPS II score, mean±SD	55.3±20.6	51.9±17.4	0.5
SOFA score at intubation. median [IQR]	5 [4–6]	5 [4–6]	0.59
Septic shock at intubation, N (%)	9 (32.1%)	3 (11%)	0.11
**Reason for ICU admission**			
Sepsis, N (%)	9 (32.1%)	3 (10.8%)	0.11
Respiratory failure, N (%)	5 (17.8%)	7 (25.0%)	0.75
Trauma, N (%)	1 (3.5%)	4 (14.3%)	0.35
Cardiogenic shock, N (%)	0 (0%)	0 (0%)	-
Neurologic disease, N (%)	1 (3.5%)	8 (28.6%)	**0.03**
Cardiac Arrest, N (%)	2 (7.1%)	1 (3.5%)	1
Others, N(%)^a^	10 (35.7%)	5 (17.9%)	0.23
**Ventilatory settings**			
PSV, N (%)	17 (60.7)	16 (57.1)	0.74
ACV, N (%)	11 (39.3)	12 (42.9)	0.75
Ppeak, cmH20	23.2±11.4	28.3±14.3	0.22
PEEP, cmH20	5.7±1.5	6.4±3.8	0.22
PaO2, mmHg	90.1±56.5	96±69.3	0.42
Respiratory rate, breaths/min	18.9 ±4.7	16.5 ±6.2	**0.03**

Data are shown as mean±SD, until otherwise indicated

^*a*^ Acute Renal failure, Hemorrhagic shock, Liver failure

*Ag* Anapnogurd; *COPD* chronic obstructive pulmonary disease; *SAPS II* Simplified Acute Physiology Score, *SOFA* Sequential Organ Failure Assessment, *ARDS* acute respiratory distress syndrome, *PSV* pressure-support ventilation, *ACV* assisted-controlled ventilation, *Ppeak* peak inspiratory pressure, PEEP positive end-expiratory pressure, *PaO2* arterial partial pressure of oxygen, *IQR* interquartile range

### Adverse events, P_cuff_ control and subglottic secretions evacuation

The median duration on the AG 100 system was 4 [1–9] days / 96 [24–216] hours. No AE/SAE potentially related to under or over inflation of the endotracheal tube (ETT) cuff or suction dysfunction was detected in either the AG or control group (Table 2). Eighteen patients were not studied with post-extubation bronchoscopy (i.e. deaths while intubated, unscheduled extubations, urgent tracheal reintubations). Among 38 patients undergoing tracheal bronchoscopic evaluation, five showed mucosa oedema (three in the AG group and two in the control group, *p* = 0.65). Eleven patients were reliably evaluable for post-extubation hoarseness and throat pain (i.e breathing and fully cooperative) and no differences were detected between the two groups (*p* = 0.65 and *p* = 0.7, respectively*)*. Among the 31 (10 physicians and 21 nurses) respondents to the questionnaire, the satisfaction level was high with the highest scores (4-5/5) being seen when referencing safety, P_cuff_ control, and subglottic secretions evacuation (Table 2).

**Table 2:** Outcome measures in the Anapnoguard 100 and control groups.

	All (n = 56)	AG 100 Group (*n* = 28)	Control Group (*n* = 28)			*P* value
**Primary Outcome**						
Device related AEs	0	0	0			-
**Secondary Outcomes**						
Post-extubation throat pain, median [IQR], (11pts) ^a^	0 [0–3]	0 [0–2]		0 [0–3]	0.7	
Post-extubation hoarseness, (11 pts)^a^	6 (54.5)	3 (42.9)		3 (75)	0.55	
Post-extubation tracheal mucosa edema, (38 pts)^b^	5 (13.2)	3 (16.7)		2 (10)	0.65	
Satisfaction questionnaire score, mean±SD, (31 respondents)^c^	-	4.1±0.8		-	-	
Pcuff, cmH20, mean±SD	27.2±4.2	29.1±3.2		25.2±4.4	**<0.001**	
Total subglottic secretions drained, ml, median, IQR^d^	150 [52–294.5]	192.0 [64–413]		150 [50–200]	0.19	
VAP, total^e^	14 (26.9)	4 (14.8)		10 (40)	0.06	
Early-onset VAP	4 (7.7)	1 (3.7)		3 (12)	0.34	
Late-onset VAP	10 (19.2)	3 (11.1)		7 (28)	0.17	
Duration of MV, days, median [IQR]	4.9 [3–13]	4 [2–13]		5 [3–13.5]	0.1	
Connection to AG 100 system, days, median [IQR]	-	4 [1–9]		-	-	
Connection to AG 100 system, hours, median [IQR]	-	96 [24–216]		-	-	
Length of ICU stay, days, median [IQR]	10 [5–29.1]	13 [6–25]		10 [4.5–17.5]	0.2	
Extubation	30 (53.6)	15 (53.6)		15 (53.6)	0.79	
Tracheostomy	17 (29.3)	6 (21.4%)		11 (39.3%)	0.15	
ICU deaths	9 (16.1)	7 (25)		2 (7.1)	0.14	

Data are shown as N (%), until otherwise indicated

^*a*^ Post-extubation throat pain and hoarseness were assessable in 11 extubated patients: 7 in the AG group and 4 in the Control Group. See the text for further details.

^*b*^ Tracheal mucosa oedema was evaluated by bronchoscope in 38 patients: 18 in the AG 100 Group and 20 in the Control Group

^*c*^ Satisfaction level was calculated from 31 respondents among the ICU staff

^*d*^ The amount of daily subglottic secretions drained was evaluated in 43 patients: 28 in the Control Group and 15 in the AG Group (in 13 out of 28 patients in the AG group, the estimation of SS drained net volume [i.e. total suction fluids—total rinsing saline], based on the data logger of the AG system, was not accurate and therefore excluded from the analysis).

^*e*^ VAP occurrence was evaluated in 52 patients: 27 in the AG Group and 25 in the Control Group.

*AE* adverse events; *Pcuff* intracuff pressure; *VAP* ventilator-associated pneumonia; *MV* mechanical ventilation; *ICU* Intensive Care Unit; *AG* Anapnoguard; *IQR* interquartile range

**Fig 2:**
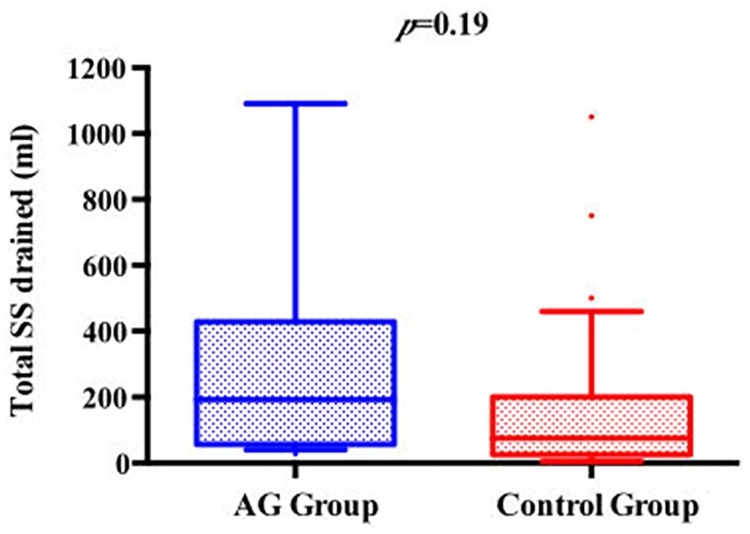
Comparison between AG group and control group according to total SS drained.

**Fig 3:**
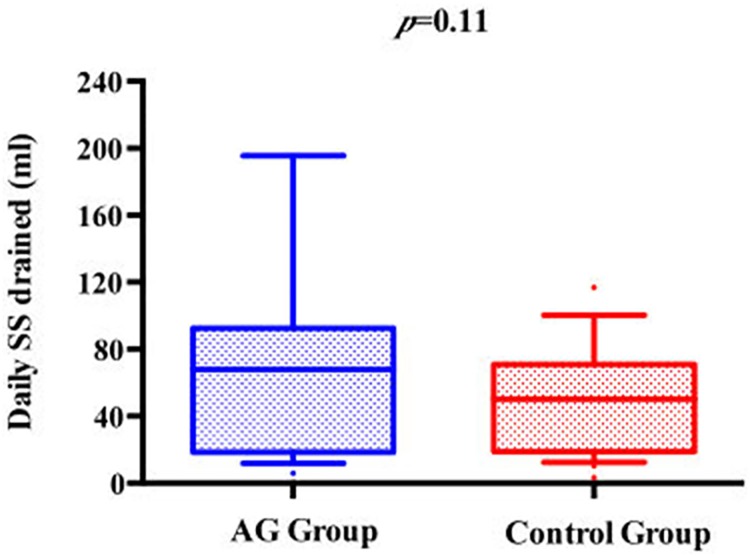
Comparison between AG group and control group according to daily SS drained. *AG*, Anapnoguard; *P*_*cuff*_, Cuff Pressure; *SS*, Subglottic Secretions.

We observed a lower mean P_cuff_ among patients from the control group compared to the AG group (25.2±4.4 cmH_2_0 vs. 29.1±3.2 cmH_2_0; p<0.01). Conversely, the proportion of P_cuff_ determinations within the predefined safety target was higher in the AG group compared with controls (97.3% vs. 71%; p<0.01). The total and the daily amounts of SS evacuated were higher in the AG group compared with controls (192 [64–413] vs. 150 [50–200], *p* = 0.19; 67.8 [20–89] vs. 50 [19–62], *p* = 0.11, respectively) (Figs 2 and 3).

### Clinical and microbiological outcomes

**Fig 4:**
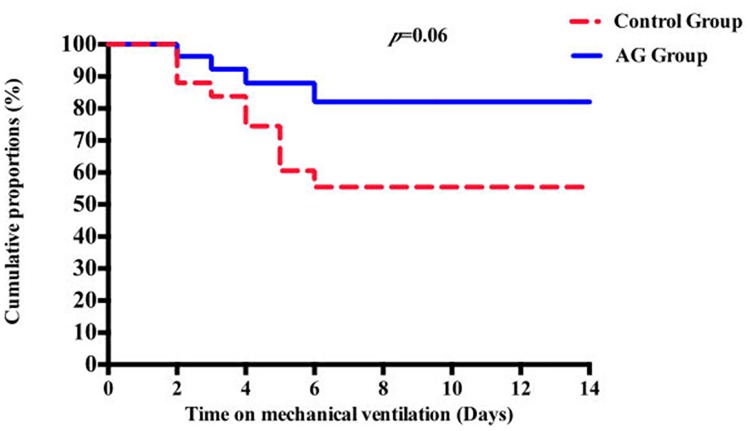
Cumulative rates of patients remaining free of VAP in the AG group and control group, using the Kaplan-Meier method. *VAP*, Ventilator-Associated Pneumonia; *AG*, Anapnoguard.

Fifty-two out of 56 enrolled patients underwent complete screening for pulmonary infections detection and were full compliant with VAP prevention strategies. Four subjects were dropped out from VAP analysis due to screening failure (clinical suspicion of pneumonia at intubation, which was microbiologically confirmed after few days); one in the AG group and three in the control group. The rates of microbiologically confirmed VAP was 14.8% in the AG group and 40% in controls, for a relative risk reduction of 63%. Survival curve analysis showed a trend to a reduced VAP risk associated with AG 100 use (*p* = 0.06) (Table 2, Fig 4, see S1 File for further details).

Twenty-four bacteria were detected at a concentration of at least 10^4^ colony forming units from the broncholaveolar lavage (BAL) fluid of 14 patients with VAP (S1 File, S1 Table). The rates of polymicrobial VAP and of positive microbiological respiratory samples were significantly higher in the control group (70% vs. 25%, *p* = 0.03 and 76% vs. 18.5%, *p*<0.01, respectively).

The tracheostomy rate, duration of mechanical ventilation, and length of ICU stay were not significantly different between the two populations. ICU mortality was 25% in patients connected to the AG system and 7.1% in the controls (*p* = 0.14) (Table 2).

## Discussion

In this study no SAE or AE was observed in either cohort. The rates of post-extubation throat pain, hoarseness and tracheal mucosa oedema were similar in both groups. The AG 100 system provided an accurate control of ETT P_cuff_ and an effective drainage of subglottic secretions, contributing to the observed trend toward the reduction of VAP incidence.

During last decades, the wide distribution of high-volume low-pressure (Hi-Lo) ETTs, helping to avoid ETT cuff overinflation, has significantly reduced the frequency of ischemic tracheal lesions[19]. However, in a randomized controlled animal study where the efficacy of a pneumatic device for P_cuff_ management was tested, no differences were identified in the prevalence of tracheal mucosa lesions between the two animal groups[20]. It is also known that both automated intermittent and continuous subglottic aspirations may be ineffective and at times injurious to the tracheal mucosa in patients with low secretions production[8, 21]. This issue was addressed in an animal investigation conducted by *Berra et al*., where 14 sheep, intubated with Hi-Lo ETT and connected to a CASS system, were found to show tracheal mucosal injuries of different degrees of severity at the level of the suction port[22].

In our study the rate of post-extubation tracheal mucosa oedema was quite low (13.2%) and it did not differ between the groups. Unfortunately, only a part of enrolled patients were evaluable for throat pain and hoarseness, but no cases of post-extubation laryngeal obstruction or stridor were documented. These results are in line with the majority of clinical studies addressing the clinical advantages of Hi-Lo ETTs use, in conjunction with strict P_euff_ control and SS drainage[23, 24], thus confirming the safety of Anapnoguard 100 system/ETT for the management of critically ill ventilated patients.

**Fig 5:**
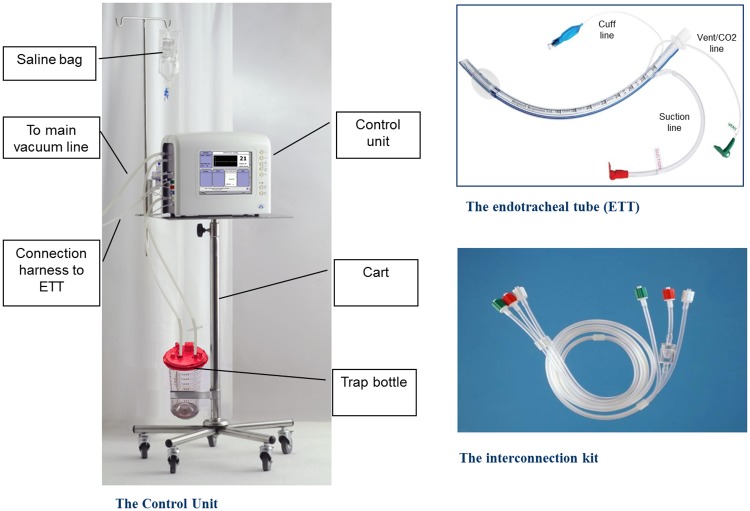
AnapnoGuard 100 system overview.

**Fig 6:**
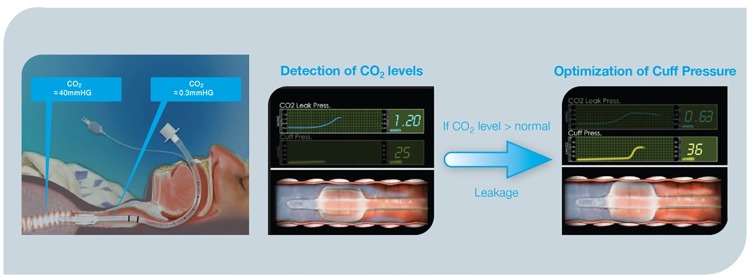
Automatic endotracheal tube cuff pressure closed loop adjustment.

Automatic continuous control of P_euff_ is now considered a milestone in the optimization of mechanically ventilated patient management and it is not surprising that the AG 100 was more effective than intermittent manual control to maintain P_cuff_ within the predefined safety range[11, 25–27]. Traditional systems, while being effective in maintaining P_cuff_ value at a predefined target, are not able to identify the optimal P_cuff_ for the individual patient in terms of microaspiration prevention and tracheal mucosa protection. Conversely, the innovative AG 100 technology, by the detection of CO_2_ levels above the ETT cuff, controls P_cuff_ to the minimum value required to seal the trachea, avoiding excessive tracheal mucosa compression while minimizing the occurrence of aspiration (Figs 5 and 6).

Of note, it is interesting that study showed a mean P_cuff_ in the AG group that was significantly higher than controls. Giving the homogenous baseline characteristics of the two study populations in terms of ventilation modes and airway pressures, it may be argued that slightly higher P_cuff_ values than commonly expected were needed to effectively and safely seal the trachea[28]. However position changes and variations in PEEP levels should be carefully evaluated in such comparisons.

**Fig 7:**
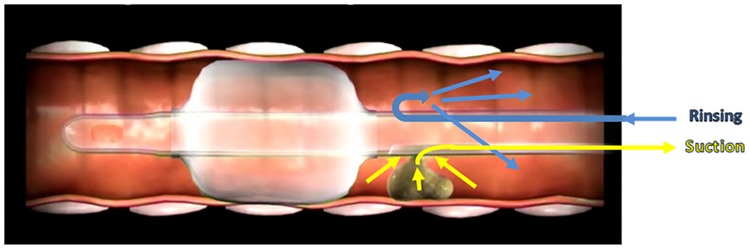
Subglottic secretions drainage based on rinsing and suctioning.

Up to now, many authors have described the efficacy of either manually intermittent or automatic continuous drainage of patients’ secretions from the subglottic space, using modified ETTs with an additional dorsal suctioning lumen[29, 30]. Avoiding the aspiration of subglottic secretions has been shown to prevent the migration of potentially pathogen bacteria (PPM) from aerodigestive tract to the lower airways, thus reducing the risk of pulmonary infections. Even though documenting the clinical benefits of SSD in preventing microaspiration of secretions from the subglottic space, the majority of existing studies do not provide details about total or daily collected volume nor the occurrence of suction line obstruction. In our study, both the overall and daily volumes of SS drained were higher in the AG group compared with controls, and this result may be interpreted in light of the characteristics of AG 100 suctioning system which, in association with the SS drainage, provides a controlled rinsing of the subglottic space with normal saline solution, allowing the dilution and improved clearance of the secretions (Fig 7).

A clear trend to a lower incidence of VAP episodes was observed in patients randomized to AG group. This observation is supported by a significant lower rate of polymicrobial infections and microbiologically positive respiratory samples. Although the trial was not designed for such a clinical endpoint, the result may be analysed on the basis of the above mentioned technical properties of this new machine. The strict P_cuff_ control and the effective subglottic secretions drainage observed in the AG group was likely to have contributed to a reduction in the amount and frequency of microaspirations from the oropharynx into the lower respiratory tract, thus influencing the pathophysiology of VAP development. The two cohorts were not fully comparable in terms of ETT cuff characteristics: AnapnoGuard ETT are provided with a ellipsoidal thin wall PU cuff, while the TaperGuard Evac ETT with a PVC conical cuff. Bench studies have suggested that the use of polyurethane and conical shaped cuffs may significantly influence the occurrence of microaspirations, optimizing tracheal seal[31–34]. In our study, all the patients evaluated for VAP analysis underwent the same preventive measures including P_cuff_ control and SSD, so such differences in ETT cuffs characteristics may have less influenced the observed between-group difference in VAP rate.

Our study has several strongpoints, but also some limitations. First, the study was unblinded and monocentric, so the results, including the satisfaction scores, may not be generalized to other settings with different microbiological patterns and VAP preventive bundles. Second, P_cuff_ in the control group was not continuously monitored and ETTs’ size was not identical in the two groups (7.5–8 in the CG and 8 in the AG group). These aspects may have influenced the observed significant difference in terms of mean P_cuff_ and volume of subglottic secretions drained. Further we did not compare both groups with a standard ETT with PVC cuff and without subglottic suction. Finally, the study did not have a third arm as control group and it was not designed to identify differences in terms of VAP rate and respiratory tract microbiological colonization, so all the clinical results regarding these aspects may represent only a proof of concept.

## Conclusion

This randomized trial showed that, in ICU critically ill mechanically ventilated patients, the use of AG ETT connected with the AG 100 system is safe and effective in terms of P_cuff_ control and evacuation of secretion from the subglottic space. The promising role of this new technology as a tool to reduce the incidence of VAP needs to be confirmed in a future, adequately powered, randomized controlled trial.

## Supporting information

S1 File Additional information. (DOCX)

S2 File CONSORT 2010 checklist. (DOC)

S3 File Protocol. (DOC)
