# Supplementary material for: CO2 driven endotracheal tube cuff control in critically ill patients: A randomized controlled study
Source: PLoS One. 2017 May 11;12(5):e0175476. doi: 10.1371/journal.pone.0175476 (PMC5426597; doi:10.1371/journal.pone.0175476)
Supplement: S1 File — (DOCX) [file pone.0175476.s001.docx]

**Additional File**

**Methods**

**Study population and Randomization**

- Device description: The AnapnoGuard 100 system is designed to provide precise and objective measurements to determine the optimal cuff pressure required for sealing the trachea at the lowest possible pressure. The system also provides controlled and effective evacuation of subglottic secretions from above the cuff. The cuff pressure control is based on the existence of high levels of Carbon Dioxide (CO2) in the exhaled air from the lungs in comparison to its concentration in the room (40mmHg vs. 0.3mmHg respectively) (eFigure 2, eFigure 3). The system controls the cuff pressure to be at the minimum pressure required for sealing of the trachea. When the cuff seals the trachea, the level of CO2 above the cuff is similar to its room/environment level. However, if the cuff does not completely seal the trachea, air with high CO2 levels will leak from the lungs into the subglottic area, resulting in higher CO2 measurements above the cuff. The CO2 level above the cuff is sampled through a dedicated lumen in the AnapnoGuard endotracheal tube and is analyzed by a CO2 analyzer embedded inside the AnapnoGuard control unit. The system also performs an adjustable and effective evacuation/suction of secretions from above the cuff. In order to dilute and clear the secretions, the system performs controlled rinsing of the subglottic volume with Saline solution, simultaneously with the suction operation. When powered ON, the system performs a self-test procedure and a safety procedure followed by a routine sequence of sub procedures. Sustain procedure is performed for 20min (default which can be set differently by the user): during this time the cuff pressure is being kept constant (continuously, closed-loop controlled). Every 2 min (default, or other user preset) a short drain is performed and CO2 measurement is taken. In case the CO2 level above a threshold is detected, the system increases the cuff pressure according to leak level and switches ("jumps to") cuff seal Procedure. At the end of the sustain procedure, a drain procedure (suction) is performed to drain the subglottic space above the cuff in order to allow free sampling of air (to detect CO2) from above the cuff. At the end of the drain procedure a cuff seal procedure is performed. Air sample from above the cuff is sucked through the VENT/CO2 lumen into the CO2 analyser. If the measured CO2 level is above a threshold, indicating a leak between the cuff and the trachea walls, the system inflates the cuff accordingly. This will be repeated until the CO2 level above the cuff is reduced under the threshold value. If the measured CO2 level is below the threshold, indicating proper sealing of the trachea, the system will gradually reduce the cuff pressure by 1mmHg per cycle, while ensuring that the seal is still kept (be follow up CO2 measurement). If the VENT/CO2 line is occluded, the system tries again to read from the VENT line and if occlusion is still detected, the system alternates to read CO2 through the SUCTION lumen. The weaning mode is intended for operating when the patient is under low sedation /semi- or fully awake (usually at the stage of weaning from ventilation). While in weaning mode, the system maintains the cuff pressure constant, (leakage detection is disabled) and performs short suctions (every 2 min.) alternating between the suction and vent lumens (venting and saline rinsing are disabled). Weaning mode was activated during spontaneous breathing trials which were performed, whenever possible, on daily basis and whose duration was usually few hours at maximum.

**Results**

- Main reasons for exclusion were the suspicion of pneumonia or lung contusion on chest X ray (62.5%) and the presence of facial/neck trauma (20.5%). Among 60 enrolled patients four were not included in the analysis (two per each group): one was withdrawn due to machine technical dysfunction (AG group) and three to screening failures (evidence of pneumonia at intubation). No patients were lost at the follow-up.
- The cumulative incidence was 28.8 VAP episodes per 1,000 MV days in the AG group and 76.3 VAP episodes per 1,000 MV days in the CG (p<0.01). No differences were observed in terms of early or late presentation (≤/≥ 96 hours) but the cumulative incidence of late VAP was lower in the AG group (22.1 episodes per 1,000 MV days vs. 56.5 per 1,000 MV days (p<0.001)
- The durations of antibiotic therapy (overall and during mechanical ventilation with the study tubes) were similar in the two groups (8 days [4.25-`5.75] in the AG group vs. 7.5 days [2-13] in the CG, *p*=0.25; 4 days [2-7.75] in the AG group vs. 3 days [1-5] in the CG, *p*=0.13).
- Microbiological details of isolated germs are shown in eTable 1

**Primary Endpoint (Device related Adverse Events [AE])**

- Potential Adverse Events that may occur during the study in both the AnapnoGuard 100 and control groups and related to hyper/hypo inflation of the endotracheal tube (ETT) cuff and suction dysfunction. The AE are listed in the table below:

| **Potential Adverse Events** | | **Severity** |
| --- | --- | --- |
| Over inflation of the cuff resulting in: | Hoarseness / Throat pain | Mild |
|  | Tracheal Stenosis | Moderate - Severe |
|  | Tracheal rupture | Severe |
| Under inflation of the cuff resulting in: | Leakage detected by ventilator | Mild |
|  | Decrease in saturation | Mild - Moderate |
|  | Apnea | Severe |
| Suction Dysfunction resulting in: | Tracheal Edema | Mild |
|  | Mucosal Injury | Mild - Moderate |
|  | Tracheal Ulcerations | Moderate |

## Definitions Used in This Study to Determine Severity of Adverse Event (AE): *Mild* (event that is noticeable but transient, requires no treatment, and does not interfere with the subject’s daily activities). *Moderate* (sign or symptom which may be ameliorated by simple therapeutic measures; may interfere with usual activity). *Severe* (signs or symptoms that are intense or debilitating and that interfere with usual activities. Recovery is usually aided by therapeutic measures and the discontinuation of the study device may be required).

## Definition of Serious Adverse Event (SAE): Any adverse event experience that results in any of the following outcomes: Death; a life threatening event; requires or prolongs inpatient hospitalization or rehospitalisation; persistent or significant disability/incapacity; medical or surgical intervention to avoid impairment of body function.

## Relationship of an AE/SAE to the Study Device/Procedure:

- Definite: The adverse event is clearly related to the investigational treatment.
- Probable: The adverse event is likely related to the investigational treatment.
- Possible: The adverse event may be related to the investigational treatment.
- Unlikely: The adverse event is doubtfully related to the investigation treatment.
- Unrelated: The adverse event is clearly NOT related to the investigational agent(s).
- Mechanical complications rate due to ETT over inflation evaluated during the 48 hours post-extubation: i.e. throat pain level assessed by visual analogic scale questionnaire, hoarseness occurrence, tracheal mucosa lesions as detected by fiberoptic analysis and based on the presence of hyperemia/oedema, ischemia, ulcer and tracheal rupture.

*Data Collection and Definitions*

- Collected data: demographic characteristics, ICU admission diagnosis, comorbidities, vital signs, ventilator settings, medical treatments, surgical/radiographic procedures, laboratory findings, microbiological and clinical outcomes and administered medications
- VAP prevention bundle adopted in the unit: semirecumbent body position, daily sedation vacation and weaning trial (when clinically appropriate), oral washing with 0.12% chlorhexidine digluconate, use of positive end-expiratory pressure ≥ 5 cmH20. Ventilator circuits were not routinely changed and selective digestive decontamination was not performed.
- VAP diagnostic criteria: Tracheal intubation and mechanical ventilation ≥ 48 h plus new or progressive radiological pulmonary infiltrate together with at least two of the following: 1) temperature > 38°C or < 36°C; 2) leukocytosis > 12,000/mm3 or leucopoenia < 4,000/mm3 ; or 3) purulent respiratory secretions. All episodes were microbiologically confirmed by quantitative cultures of bronchoalveolar lavage (≥ 104 cfu/ml). Infection onset coincided with the collection date of the first microbiological sample culture yielding the study isolate (index culture). VAP was defined early or late according to the 96 hours threshold value.
- All clinical outcomes and radiographs were independently evaluated by two physicians (GDP and MSV) and when judgements were discordant (less than 10%), the data were reassessed with the senior investigator (MA), reaching a consensus decision.
- Satisfaction questionnaire for physicians and nurses:

| **AnapnoGuard 100 - Clinical Study**  **Satisfaction Questionnaire** | | |
| --- | --- | --- |
| *Please* ***circle*** *the most appropriate answer for each question according to the following scale:*   1. **Strongly disagree** 2. Disagree 3. Neither agree or disagree 4. Agree 5. **Strongly agree**   ***Space is provided for comments.*** | | **Scale** |
| 1. **I’m satisfied with the cuff pressure control achieved by the AnapnoGuard 100 system during mechanical ventilation.**   If you choose 1 or 2 , please explain: | | 1 2 3 4 5 |
| 1. **I’m satisfied with the effectiveness of subglottic secretion evacuation achieved by the AnapnoGuard 100 system during mechanical ventilation**.   If you choose 1 or 2, please explain: | | 1 2 3 4 5 |
| 1. **I’m satisfied with the safety of using the AnapnoGuard 100 system during mechanical ventilation.**   If you choose 1 or 2, please explain: | | 1 2 3 4 5 |
| 1. **I think that using the AnapnoGuard 100 system** **improves the treatment of ventilated patients in the ICU**   If you choose 1 or 2, please explain: | | 1 2 3 4 5 |
| 1. **I’m satisfied with the ease of operating the AnapnoGuard 100 System during mechanical ventilation.**   If you choose 1 or 2, please explain: | | 1 2 3 4 5 |
| 1. **Overall I’m very satisfied with the use of the AnapnoGuard 100 system**   If you choose 1 or 2, please explain: | | 1 2 3 4 5 |
| 1. **I intend to use the AnapnoGuard 100 system as standard of care in my ICU for mechanically ventilated patients**.   If you choose 1 or 2, please explain: | | 1 2 3 4 5 |
| 1. **I will recommend my colleagues to use the AnapnoGuard 100 system for mechanically ventilated patients**   If you choose 1 or 2, please explain: | | 1 2 3 4 5 |
| Any comments or recommendations?  ………………………………………………………………………………………………………………………………………………………………………………………………………………………………………………………………………………………………………………………………………………………………………………………………………………………………………………………………………………………………………………………………………………………………………………………………………………………………………………………………………………………………………………………………………………………………………… | | |
| **Physician Name (capital letters)** |  | |
| **Physician Position** |  | |
| **Name of hospital and department** |  | |
| **Signature** |  | |
| **Date (DD/MMM/YY)** | __________/_____________/___________ | |

**Statistical analysis**

All statistical analyses were performed using the Intercooled Stata program, version 11(StatCorpLP). Kolmogorov-Smirnov test was used to value the variables distribution. The data with a non-Normal distribution were assessed with Mann-Whitney test and the median and selected centiles (25th-75th) value was given. The data with a Normal distribution were assessed with Student’s test. Categorical variables are presented as proportions and were analyzed with the use of the chi-square test or Fisher’s exact test, as appropriate. A P value <0.05 was considered significant. MedCalc® Software (v. 12.2.1, MariaKerke, Belgium) was used for all statistical analyses. Graphing of data was undertaken using Prism version 6.0 for Windows (graphPad Software, San Diego, CA).

**Microbiology analysis**

Isolates were identified by matrix-assisted laser desorption ionization-time-of-flight (MALDITOF) mass spectrometry (MALDI Biotyper, Bruker Daltonics GmbH, Leipzig, Germany). The in vitro susceptibility of the isolates was assessed with the Vitek 2 system (bioMérieux, Marcy l’Etoile, France) or the Sensititre broth microdilution method (Trek Diagnostic Systems, Cleveland, OH). Results were interpreted in accordance with the European Committee on Antimicrobial Susceptibility Testing (EUCAST) clinical breakpoints.

**S Table 1** Microbiological profile of the 14 patients with VAP

|  | **No. (%) of microbiological isolates** | |  |
| --- | --- | --- | --- |
| **Microorganism type** | **AG 100 Group**  (n=5) | **Control Group**  (n=19) | ***P* value** |
| ***Gram-positive bacteria*** | 1 (20) | 5 (26.3) | 1 |
| *Streptococcus pyogenes* | 0 | 1 (5.6) | - |
| *Staphylococcus spp.* | 1 (20) | 4 (21.1) | 1 |
| ***Gram-negative bacteria*** | 4 (80) | 14 (77.8) | 1 |
| *Acinetobacter baumannii* | 0 | 1 (5.6) | - |
| *Citrobacter freundii* | 0 | 1 (5.6) | - |
| *Enterobacter spp.* | 1 (20) | 2 (7.1) | 0.5 |
| *Escherichia coli* | 0 | 1 (5.6) | - |
| *Haemophilus influenzae* | 0 | 4 (22.2) | - |
| *Klebsiella spp.* | 1 (20) | 3 (21.4) | 1 |
| *Pseudomonas aeruginosa* | 2 (40) | 1 (5.6) | 0.1 |
| *Serratia marcescens* | 0 | 1 (5.6) | - |

data are shown as N (%)

Multi-Drug Resistance: 2 methicillin-resistant *Staphylococcus aureus*; 2 carbapenem-resistant *Klebsiella pneumoniae*; 1 carbapenem-resistant *Acinetobacter baumannii*; 1 carbapenem-resistant *Pseudomonas aeruginosa*

**Legend**

*AG* Anapnoguard; *VAP* ventilator-associated pneumonia
