# Supplementary material for: CO2 driven endotracheal tube cuff control in critically ill patients: A randomized controlled study
Source: PLoS One. 2017 May 11;12(5):e0175476. doi: 10.1371/journal.pone.0175476 (PMC5426597; doi:10.1371/journal.pone.0175476)
Supplement: S3 File — (DOC) [file pone.0175476.s003.doc]

**HST- AG ­– 04**

**The safety of using THE AnapnoGuard 100 system in intubated critical Care patients**

**Clinical Investigation Plan (CIP)**

**March 26, 2012**

**Version 1.1**

**Content**

[Time Table 4](#__RefHeading___Toc298004216)

[Study Synopsis 5](#__RefHeading___Toc298004217)

[1. Clinical background 9](#__RefHeading___Toc298004218)

[2. STUDY OBJECTIVES AND PURPOSE 11](#__RefHeading___Toc298004219)

[2.1 Clinical Trial Design 11](#__RefHeading___Toc298004220)

[2.2 Primary Objective 11](#__RefHeading___Toc298004221)

[2.3 Secondary Objective 11](#__RefHeading___Toc298004222)

[3. ENDPOINT PARAMETERS 11](#__RefHeading___Toc298004223)

[3.1 Primary Endpoint Parameter 11](#__RefHeading___Toc298004224)

[3.2 Secondary Endpoint Parameters 12](#__RefHeading___Toc298004225)

[4. Selection and Withdrawal of Subjects 12](#__RefHeading___Toc298004226)

[4.1 Source of Subjects and Enrollment 12](#__RefHeading___Toc298004227)

[4.2 Eligibility Criteria 13](#__RefHeading___Toc298004228)

[5. MATERIALS 14](#__RefHeading___Toc298004231)

[5.1 AnapnoGuard 100 system Intended use 14](#__RefHeading___Toc298004232)

[5.1.1 Identification of the Investigational Medical Devices 15](#__RefHeading___Toc298004233)

[5.2 CASS system + Covidien Taper Guard Evac ETTs 15](#__RefHeading___Toc298004234)

[6. STUDY PROCEDURES - METHODS 15](#__RefHeading___Toc298004235)

[6.1 Informed Consent Procedure 15](#__RefHeading___Toc298004236)

[6.2 Study Time Line 17](#__RefHeading___Toc298004237)

[7. DeVICE MAINTENENCE AND DISPOSAL 20](#__RefHeading___Toc298004242)

[8. Statistical Consideration 20](#__RefHeading___Toc298004243)

[8.1 Safety Analysis Set 20](#__RefHeading___Toc298004244)

[8.2 Sample Size considerations 20](#__RefHeading___Toc298004245)

[8.3 Statistical Analysis 20](#__RefHeading___Toc298004245)

[8.4 Statistical Methods 22](#__RefHeading___Toc298004248)

[9. ADVERSE EVENTS CONSIDERATION 22](#__RefHeading___Toc298004249)

[9.1 Definitions Used in This Study to Determine Severity of Adverse Event (AE) 22](#__RefHeading___Toc298004250)

[9.2 Definition of Serious Adverse Event (SAE) 22](#__RefHeading___Toc298004251)

[9.3 Guidelines to Determine the Relationship of an AE/SAE to the Study Device/Procedure 23](#__RefHeading___Toc298004252)

[9.4 AE Regulatory Reporting Procedures 23](#__RefHeading___Toc298004253)

[9.5 Adverse Events Reported by the Sponsor (IND Safety Reports) 24](#__RefHeading___Toc298004254)

[9.6 ist of Serious Adverse Events 24](#__RefHeading___Toc298004255)

[9.7 Clinical Management of Study Related Adverse Events 25](#__RefHeading___Toc298004256)

[9.8 Product Complaints 26](#__RefHeading___Toc298004257)

[9.9 Criteria for subject’s Study Termination 26](#__RefHeading___Toc298004258)

[10. Study Monitoring 26](#__RefHeading___Toc298004259)

[10.1 Study Records/Source Document Inspection 26](#__RefHeading___Toc298004260)

[10.2 Monitors 26](#__RefHeading___Toc298004261)

[10.3 Monitoring Plan 27](#__RefHeading___Toc298004262)

[11. Confidentiality/Publication of Study Results 28](#__RefHeading___Toc298004263)

[12. Record Keeping 29](#__RefHeading___Toc298004264)

[12.1 Source Documents 29](#__RefHeading___Toc298004265)

[12.2 Data Collection Method 30](#__RefHeading___Toc298004266)

[13. REGULATORY OBLIGATIONS 30](#__RefHeading___Toc298004267)

[13.1 Sponsor's Obligations 30](#__RefHeading___Toc298004268)

[13.2 Investigator's Obligations 31](#__RefHeading___Toc298004270)

[14. Study Completion 32](#__RefHeading___Toc298004273)

[15. Protocol Deviations and Exceptions 32](#__RefHeading___Toc298004274)

[16. Removal of Subjects from the Study 33](#__RefHeading___Toc298004275)

[17. Ethical Conduct of the Study 33](#__RefHeading___Toc298004276)

[18. references 33](#__RefHeading___Toc298004276)

**Time Table**

|  | **Screening** | **Connection to AnapnoGuard System** | **Mechanically Ventilation**  **Period** | **After AnapnoGuard Disconnection** | **Post Clearance Follow-up Phone Information** |
| --- | --- | --- | --- | --- | --- |
| **Time line** | **During hospitalization in ICU** | **Up to 6 h from intubation initiation** | **Daily evaluation** | **48h post extubation** | **1 month following extubation** |
| **Informed consent** | x |  |  |  |  |
| **Inclusion criteria** | x |  |  |  |  |
| **Medical history** | x |  |  |  |  |
| **Chest x-ray** | x | X | x* | x |  |
| **Blood tests** | x | X | x | x |  |
| **Vital signs** | x | X | x | x |  |
| **Trap bottle secretions***** |  |  | x |  |  |
| **Oxygen saturation** | x | x | x | x |  |
| **Mechanical complication assessment**** |  |  |  | x |  |
| **Subject’s general condition** |  |  | x | x | x |
| **AE and SAE** |  | x | x | x |  |
| **Study deviation** |  | x | x | x |  |
| **Final statistical analysis** |  |  |  | x |  |
| **Study exit** |  |  |  | x |  |

* Chest X-ray will be done at baseline, 48 hours after intubation and whenever there is clinical suspicion for VAP

** Mechanical complications assessment will include: spirometry, fiber optic in selected ICUs and questionnaire regarding throat pain and hoarseness.

***Recording level of trap bottle secretions 3 times a day (per shift). Records – Images and or electronic record

**Study Synopsis**

| **Study title** | **The safety of using AnapnoGuard 100 in intubated critical Care patients** |
| --- | --- |
| **Study number** | Protocol # **HST- AG­– 04**;Version 1.1 |
| **Sponsor** | Hospitech Respiration Ltd. |
| **Study design** | Prospective, single - center, double-arm, randomized-controlled open label, safety study. |
| **Recruitment duration** | Estimated recruitment duration: approximately 12 months. Study duration for each subject depends upon mechanical ventilation duration, which will be defined by subject’s general condition. |
| **Study site** | The study will be conducted at the ICU of the Università Cattolica del Sacro A Gemelli University Hospital, Rome, Italy. in Rome. Additional ICU may be added. |
| **Principal Investigator** | Professor Massimo Antonelli, Director of the institute of Anaesthesiology and Intensive care. |
| **Investigational product** | Intended Use/Indications for Use:  ***AnapnoGuard™ 100 Respiratory Guard*** System is intended for airway management by oral/nasal intubation while providing continuous endotracheal cuff pressure control using non-invasive measurement and monitoring of carbon dioxide concentration in the subglottic space and evacuation of secretions from above the endotracheal tube’s cuff.  The system is comprised of the following main three components:   1. AnapnoGuard 100 control unit 2. Single use AnapnoGuard endotracheal tube with two suction lumens and a vent lumen 3. Single use AnapnoGuard interface harness and filters kit |
| **Patient population** | Patient admitted to ICU and expected to receive mechanical ventilation for at least 12 hours. Patient will be randomly assigned to study or control group using the block randomization method. |
| **Patient selection criteria** | **Inclusion Criteria**  Subjects eligible for this clinical study must fulfill **all** of the following:   1. Age above 21 (men and woman); 2. Patient is admitted to ICU and expected to receive mechanical ventilation for more than 12 hours; 3. Absence of clear signs of pneumonia and lung contusion on chest X ray; 4. Connection of the ETT to the AnapnoGuard system less than 6 hours from intubation initiation; 5. No fever or fever from known from other non chest/lung origin;     **Exclusion Criteria**  Subjects not eligible for this research study include those that **have any** of the following:   1. Patients who had been treated with mechanical ventilation during the last 3 months; 2. Patients with facial, oropharyngeal or neck trauma 3. BMI>40 4. Pregnant women 5. Patient ventilated in prone position  Difficult intubation (defined as more than 3 intubation attempts) |
| **Primary objectives** | The study primary objective is:   - Evaluation of the safety of using the AnapnoGuard 100 system during the course of mechanical ventilation and intubation in Intensive Care Unit (ICU); |
| **Secondary objectives** | The study secondary objectives are   - Evaluate the occurrence of mechanical complication due to ETT cuff over inflation. - Physician satisfaction from AnapnoGuard 100 system |
| **Primary outcome measures** | Primary safety outcome measure in this study is:   - Rate of AE (adverse events) and SAE (serious adverse events) while using the AnapnoGuard 100 system during the course of mechanical ventilation and intubation in Intensive Care Unit (ICU). |
| **Secondary outcome measures** | - - Mechanical complication occurrence due to ETT cuff over inflation will be evaluated by the following measures during the 48 hours post extubation:   - Throat pain level, associated with the mechanical ventilation. Pain level will be assessed by VAS (visual analogue scale) Scale;   - Hoarseness occurrence   - Spirometry analysis   - Fiber optic analysis (this measure will be used in selected ICUs) - Medical staff satisfaction from AnapnoGuard 100 system will be established by designated questionnaire. |
| **Other Observation** | - Evaluation of the effect of AnapnoGuard system upon ventilator associated pneumonia (VAP) occurrence rate and time to VAP in intubated patients in intensive care units (ICU). |
| **Definition of the safety of AnapnoGuard system** | This study has primary endpoint - *rate of AE and SAE as a result of using AnapnoGuard 100 during mechanical intubation.* Thus, a subject's responsiveness is evaluated as following:   - A subject is considered a “success “on *safety* if no device related AE or SAE has occurred during intubation. |
| **Sample size** | Presentation of sample size is based on achieving 80% power overall to demonstrate that the *rate of AE and SAE as a result of using the AnapnoGuard 100 during mechanical intubation* is up to 0.20. Thus, the determination of sample size in this trial will be based on *rate of AE and SAE as a result of using AnapnoGuard 100 during mechanical intubation*.  A former study using *AnapnoGuard 100* during mechanical intubation in ICU revealed **no related AE or SAE** (all together 29 patients were studied). Consequently, we feel that 80% (of study patients with no AE or SAE during intubation) to be an appropriate clinical target for AnapnoGuard 100 success rate. Assuming a true success rate of 80%, a sample of **N = 25** will provide 80% power to show that AnapnoGuard 100 success (no AE or SAE during intubation) is at least 80%. This is based on a power analysis using the normal approximation for the binomial, with one-sided Alpha=0.05.  As control treatment is assumed to have the same success rate (at least 80% cases with no AE or SAE during intubation), same number of patients will be recruited to the control group **(N=25)**  To account for an approximate 15% dropout rate, we set a total number of patients N=**60** (for study and control groups together). |
| **Primary endpoint and study hypothesis** | For the primary endpoints we will compute Overall Proportion of Success: $\text{P(success) }=\text{ }\frac{\text{Total \# of Successes}}{\text{Total \# in Cohort}}$   The following hypotheses are specified:  The safety proportion of success (*No AE or SAE while using AnapnoGuard 100 during intubation* -π_R_):  H_0_: π_R_ < 0.80  H_1_: π_R_ > 0.80  This hypotheses will be tested by:   1. Constructing a one-sided, lower 95% confidence interval about the observed Overall Proportion of Success in the relevant cohort; 2. Examining whether the lower limit of the confidence interval is at or above the success criterion   Study success will be declared if the following is met:  Lower confidence limit of *No* *device related AE and SAE as a result of using AnapnoGuard 100 during mechanical intubation* success rate is at 0.80 or above |

# Clinical background

Appropriate endotracheal tube (ETT) cuff inflation is an important part of the management of mechanically ventilated patients (1-5). An appropriately inflated ETT cuff should achieve isolation of the lower airways, thus reducing the risk of aspiration around the cuff and possible ventilator-associated pneumonia (VAP) (5–10). However, an over-inflated cuff may cause local mechanical complications such as mucosal ischemia, ulcerations, granulomas, tracheal stenosis, and tracheoesophageal fistulae (1– 5). Consequently, appropriate pressure maintenance of the ETT cuff is recommended in the guidelines (11).

The routine management of cuff inflation consists of periodic manual checking of the Pcuff (Intra Cuff Pressure), which does not ensure the appropriate maintenance of the Pcuff, during continuous tracheal intubation (5, 12). Moreover, the manual checking of the Pcuff may cause either over inflation or over deflation of the cuff that may cause aspiration of contaminated secretions to the lower airway during the maneuver (5, 12). Several devices that provide an automatic and continuous effective control of the Pcuff have been described in the literature. Even though, the automatic devices may provide effective continuous control of the Pcuff, they do not reduce rate of mechanical complications, such as tracheal hyperemia or hemorrhages, and they do not improve the rate of VAP, ICU mortality, or hospital mortality (13, 14). The main drawback of all these devises is that they cannot predict the true optimal cuff pressure for the specific patient so the target cuff pressure is being set arbitrarily for all patients.

The AnapnoGuard system (Hospitech Respiration LTD., Petach-Tikva, Israel) is an innovative respiratory guard system that continuously monitors and controls the cuff pressure. The optimal cuff pressure is being set by measurements of CO2 levels above the cuff (15). The optimal ETT cuff pressure is the minimal pressure needed to prevent CO_2_ leakage from the lungs to the oropharynx. Since the method is non-invasive (using a dedicated line in the ET tube), the optimal cuff pressure can be determined continuously as long as the patient is intubated. In a previous study, including elective surgical patients, the cuff pressure set by anesthesiologists was accurate in only 15% of the patients (15). During surgery, a new leak was created around the ETT cuff, as measured by CO_2_, developed in 27% of the patients. This leak was attributable to variable causes such as increased peak inspiratory pressure by >5 mm Hg caused by laparoscopic surgery with abdominal gas inflation, light anesthesia or inadequate neuromuscular blockade, changes of head positions, and ETT movement during surgery. In the ICU, patients are under prolonged mechanical ventilation with daily or even hourly changes in ventilation parameters, patient position, intra-abdominal pressure and degree of anesthesia.

A pilot study was performed with the AnapnoGuard system in two general ICU departments in Bucharest, Romania. A total of 29 patients were intubated with the AnapnoGuard tube and were connected to the AnapnoGuard System. Of the 29 patients, 17 patients were connected to the system under a clinical routine practice, as the AnapnoGuard 100 is cleared for marketing thought Europe (CE cleared). 12 patients were enrolled into the study. Of the 12 patients, 9 patients eventually met all inclusion/exclusion criteria. No related adverse events were reported for all 27 patients. Physician questionnaire results indicated that the physicians were satisfied with the ease of use of the system as well as its efficacy in keeping low cuff pressure sealing the trachea while reducing mechanical pressure and reducing VAP incidence. VAP was reported in one patient (11%) ventilated by the AnapnoGuard 100 system and in seven patients (70%) in the retrospective group.

This study was designed in order to evaluate the safety of the AnapnoGuard system in ICU patients ventilated for more than 12 hours. The possible complications this study aims to address are related to inappropriate cuff pressure in patients under prolonged ventilation. The complications may include mechanical complications as a result of over inflation of the cuff and leakage as a result of under inflation of the cuff. These measures are best identified and analyzed if a patient is intubated for more than 12 hours.

# STUDY OBJECTIVES AND PURPOSE

## 2.1 Clinical Trial Design

This study is a Prospective, Single - center, Double-arm, Randomized-controlled Open label, safety study.

## Primary Objective

The primary objective of this study is to evaluate the safety of using the AnapnoGuard 100 system during the course of mechanical ventilation and intubation in Intensive Care Unit (ICU).

## Secondary Objective

- Evaluate the occurrence of mechanical complication due to ETT cuff over inflation.
- Physician satisfaction from AnapnoGuard system.

# 3. ENDPOINT PARAMETERS

## 3.1 Primary Endpoint Parameter

This study has primary endpoint - *rate of device related AE and SAE as a result of using AnapnoGuard 100 during mechanical intubation.* Thus, a subject's responsiveness is evaluated as following:

- A subject is considered a “success “on *safety* if no device related AE or SAE has occurred during intubation.

Serious adverse event are defined as adverse requiring operation and/or associated with prolongation of hospital stay. Serious adverse events occurrence will be compared to serious adverse events incidence in the control group of mechanically ventilated patients in intensive care units.

## 3.2 Secondary Endpoint Parameters

- Mechanical complication occurrence due to ETT cuff over inflation will be evaluated by the following measures during the 48h post extubation:
  - Throat pain level, associated with the mechanical ventilation. Pain level will be assessed by VAS (visual analogue scale) questionnaire;
  - Hoarseness occurrence
  - Spirometry analysis
  - Fiber optic analysis (this measure will be used in selected ICUs)
- Medical staff satisfaction from AnapnoGuard 100 system operations and performance will be established by designated questionnaire for physicians and nurses.

**3.3 Other Observations**

- Evaluation of the effect of AnapnoGuard system upon ventilator associated pneumonia (VAP) occurrence rate and time to VAP in intubated patients in intensive care units (ICU)

# 4. Selection and Withdrawal of Subjects

## 4.1 Source of Subjects and Enrollment

Male/female subjects admitted to ICU and expected to be mechanically ventilated for at least 12 hours. A minimum of 12 hours of intubation is required due to the fact that the AnapnoGuard 100 system’s intended use is for patients with prolonged ventilation with endotracheal tubes. In order to evaluate the safety of the device, the subject should be intubated for at least 12 hours.

~~Only subjects that their legally acceptable representative have signed an Informed Consent Form and meet all of the eligibility criteria listed below will be qualified for enrollment. Upon recruitment, subjects will be randomized, using the sequentially numbered opaque sealed envelopes (SNOSE) randomization method, to be treated with AnapnoGuard system (Study Group) or to be treated according to the standard ICU procedure (Control Group).~~

Male/female subjects admitted to ICU and expected to be mechanically ventilated for at least 12 hours. Subjects will be randomized to study/control group prior to enrollment in blocks of ten (the first 10 subjects (1-10) will be selected for study group; the second 10 subjects (11-20) will be selected for the control group, and so on). Subjects will be intubated with either Hospitech’s ETT or Covidien’s ETT (10-10) and only after intubation they will be screened. Only subjects that meet all of the eligibility criteria listed below and their legally acceptable representative has signed an Informed Consent Form will be qualified for enrollment.

## 4.2 Eligibility Criteria

**Inclusion Criteria**

Subjects eligible for this clinical study must fulfill all of the following:

1. Age above 21 (men and woman);
2. Patient is admitted to ICU and expected to receive mechanical ventilation for more than 12 hours;
3. Absence of clear signs of pneumonia and lung contusion on chest X ray;
4. Connection of the ETT to the AnapnoGuard system less than 6 hours from intubation initiation;
5. No fever or fever from known from other non chest/lung origin;
6. ~~Signed informed consent by subject’s legally acceptable representative~~.

**Exclusion Criteria:**

Subjects not eligible for this research study include those that have any of the following:

1. Patients who have been treated with mechanical ventilation during the last 3 months;

2. Patients with facial, oropharyngeal or neck trauma

3. BMI>40

~~4. Any chest X-ray pathology during the first 24 hours post Intubation~~

4. Pregnant women

## 5. Patient ventilated in prone position

## 6. Difficult intubation (defined as more than 3 intubation attempts)

**Subject Withdrawal Criteria**

Subjects may be withdrawn from the study if any one or more of the following events occur:

1. Subject’s legally acceptable representative has the right to decide regarding subject withdrawing from the study at any time without providing any explanation.
2. Refusal of the subject’s nearest relative to continue follow-up observations.
3. Any significant adverse event that upon the investigator opinion may be related to the AnapnoGuard system.
4. Significant protocol deviation.
5. Less than 12 hours connection to the AnapnoGuard 100 system.
6. Any chest x-ray pathology during the first 12 hours post intubation.
7. Patients who underwent re-intubation

## Handling of Withdrawals

In accordance with the current revision of the Declaration of Helsinki, a subject’s legally acceptable representative has the right to decide regarding subject withdrawing from the study, at any time, for any reason, without prejudice to the subject’s future medical care by the physician or the institution. The investigator and the sponsor also have the right to withdraw subjects from the study in the event of serious adverse events, protocol departure, or other reasons. Should a subject’s legally acceptable representative decide to withdraw, all efforts will be made to collect and report the final visit observations, and the reasons for withdrawal, as thoroughly and timely as possible.

Withdrawals will be recorded, analyzed and reported to local IRB or ethical committees.

# 5. MATERIALS

## 5.1 Study Group: AnapnoGuard 100 system

## 5.1.1 Intended use

***AnapnoGuard™ 100 Respiratory Guard*** System is intended for airway management by oral/nasal intubation while providing continuous endotracheal cuff pressure control using non-invasive measurement and monitoring of carbon dioxide concentration in the subglottic space and evacuation of secretions from above the endotracheal tube’s cuff.

The system comprises of the following main three components:

- AnapnoGuard 100 control unit
- Single use AnapnoGuard endotracheal tube with two suction lumens and a vent lumen
- Single use AnapnoGuard interface harness and filters kit

##

## 5.1.2 Identification of the Investigational Medical Devices

The investigational AnapnoGuard 100 system will be labeled for use in clinical trials only. The label includes trade name and address of the manufacturer, Serial Number and all other required information.

## 5.2 Control Group:

# The Control group will be intubated with the TaperGuard EVAC endotracheal tubes ID 8mm by Covidien. After the intubation, patients in the control group will be connected to the Boehringer CASS regulator (Continuous Aspiration of Subglottic Secretion) until extubation.

# 6. STUDY PROCEDURES - METHODS

## 6.1 Informed Consent Procedure

- - In obtaining and documenting informed consent, the investigator should comply with the applicable regulatory requirements, and should adhere to GCP, HIPAA and to the ethical principles that have their origin in the Declaration of Helsinki.
  - Prior to the beginning of the trial, the investigator should have the Ethics Committee written approval/favorable opinion of the written informed consent form and any other written information to be provided to subject’s legally acceptable representative.
  - The written Informed Consent Form (ICF) and any other written information to be provided to subjects legally acceptable representative should be revised whenever important new information becomes available that may be relevant to the subject’s consent. Any revised written ICF and written information should receive the Ethics Committee’s approval/favorable opinion in advance of use. The subject or the subject’s legally acceptable representative should be informed in a timely manner if new information becomes available that may be relevant to the subject’s willingness to continue participation in the trial. The communication of this information should be documented.
  - Neither the investigator, nor the trial staff, should coerce or unduly influence a subject’s legally acceptable representative to participate or to continue to participate in a trial.
  - The investigator, or a person designated by the investigator, should fully inform the subject’s legally acceptable representative, of all pertinent aspects of the trial including the written information given approval/favorable opinion by the Ethic Committee.
  - Before informed consent may be obtained, the investigator, or a person designated by the investigator, should provide the subject’s legally acceptable representative ample time and opportunity to inquire about details of the trial and to decide whether or not to participate in the trial. All questions about the trial should be answered to the satisfaction of the subject's legally acceptable representative.
  - ~~Prior to a subject’s participation in the trial,~~ the written ICF should be signed and personally dated by the subject's legally acceptable representative, and by the person who is authorized to conducted the informed consent discussion.
  - ~~Prior to participation in the trial,~~ the subject's legally acceptable representative should receive a copy of the signed and dated written ICF and any other written information provided to the subjects. During a subject’s participation in the trial, the subject’s legally acceptable representative should receive a copy of the signed and dated consent form updates and a copy of any amendments to the written information provided to subjects.
  - Subject's legally acceptable representative must provide informed consent and sign and date with his hand writing an ICF prior to any study related procedures being performed. The person reviewing the ICF with the subject must also sign and date the ICF. Each of the signers must sign and date in the presence of the other signer.
  - Allocated informed consent study staff will make efforts to obtain written informed consent prior to enrollment (for study patients – Before connection to the AnapnoGuard 100 system and for control patients - as soon as possible following intubation). Nevertheless, for specific cases (e.g. patients arriving at the ICU without an accompanying legal representative, there will be a 24 hour window to achieve written informed consent. The reason for enabling a 24 hour window for written ICF in the control group has to do with the fact that these patients will be treated with conventional standard of care. A 24 hour written ICF is allowed for the study group as well as for the control group since the investigational device, the AnapnoGuard 100 respiratory system, is a commercially (CE Mark) available device that is intended for non-life supporting functions and performs the following two basic operations; cuff pressure control and suctioning above the cuff.

## 6.2 Study Time Line

Study procedure will take place in the Intensive Care Unit, and will continue at least 48 hours following disconnecting from the AnapnoGuard system. Study duration for each subject depends upon mechanical ventilation duration, which will be defined by subject’s general condition (not depended upon study procedure). Altogether, study duration will be approximately 12 months, depending on subject recruitment rate.

### 6.3 Screening procedure

The following will be collected or performed before any study-specific procedures are performed:

- Ensure that all inclusion criteria are met.
- Obtain Informed Consent Form (the individual conducting the informed consent process must also sign the consent form and document it in the subject’s source record).
- Obtain blood count lab tests.
- Obtain chest X-ray.
- Obtain demographics (age, family history, and gender),
- Complete physical examination, current medications that subject is using, and medical and surgical history (anamnesis).
- Assign a sequentially ordered subject number per each site (which will specify the site and subject number). If a subject withdraws or is withdrawn from the Study or fails to undergo procedure, the subject number cannot be reassigned.
- Inform the study monitor about the subject’s recruitment and the planned date of treatment.

### 6.4 Connection to ETT, Operation and Monitoring Procedure

Following enrollment, subjects will be randomized to a study group or control group using the block randomization method. Treatment will be as follows:

- The study group patients will be treated with the AnapnoGuard 100 system and will be intubated with the AnapnoGuard's ETTs.

## The control group patients will be intubated with the TaperGuard EVAC endotracheal tubes by Covidien.

6.5 Treatment of study Group

Patients assigned to the study group, will be connected to the AnapnoGuard 100 system. The AnapnoGuard tubes (AnapnoGuard endotracheal tubes, Hospitech Respiration LTD, Petach-Tikva, Israel) will be used for intubation (ETT). Once connected to the AnapnoGuard 100 system, optimal cuff pressure will be set automatically according to CO_2_ level measured by the AnapnoGuard system in the subglottic space between the ETT cuff and the vocal cords. In addition, the AnapnoGuard system will perform programmed, Intermittent Aspiration of Subglottic Secretions below the vocal cords using the two suction lumens of the ETT. Optimal cuff pressure determination will be performed every 20 minutes (default) or according to the AnapnoGuard 100 algorithm. Cuff pressure and any change in cuff pressure will be automatically recorded by the AnapnoGuard 100 data logger system.

6.6 Treatment of control group

Cuff pressure will be set by manometer according to the physician judgment. Cuff pressure will be measured and set at least once daily. In case of using suction ET tubes, suction will be performed manually or by the use of the Boehringer 3720 CASS regulator, according to the manufacture instructions.

**6.7 Data collection**

The following parameters will be recorded with respect to the optimal cuff pressure:

- Patient position
- Ventilator parameters will be recorded (the setting will be done according to the physician clinical judgment):
- Assist vs. Control ventilation
- Pressure vs. Volume controlled ventilation
- Peak inspiratory pressure
- Peak end expiratory pressure (PEEP)
- Respiratory rate
- Vital signs will be recorded every 4 hours (blood pressure and temperature);
- Chest x-ray will be obtained on a daily basis;
- Blood test for white blood cell count and differential;
- Medication; special emphasis on antibiotic use.
- Daily recording of the amount of secretions in the AnapnoGuard trap bottle.
- Oxygen saturation;
- Oxygen concentration;
- Any interventional procedure performed by the medical staff;
- Any disconnection from the AnapnoGuard system;
- Study deviations will be documented if applicable;
- Adverse events will be managed and documented.

### 6.8 AnapnoGuard 100 System Disconnection

Prior to disconnection, during weaning from mechanical ventilation, the following will be evaluated:

- Level of sedation according to the Richmond Agitation Sedation Scale (RASS).
- Duration of the weaning process and medications usage and dosages.

After extubation, all patients will be scanned by chest x-ray.

During 48 hours following extubation, the following parameters will be used for evaluation of possible mechanical complications:

- - Throat pain level, associated with the mechanical ventilation. Pain level will be assessed by VAS (visual analogue scale) questionnaire.
  - Hoarseness occurrence
  - Spirometry analysis
  - Fiber optic analysis (this measure will be used in selected ICUs)
  - VAP Occurrence

# 7. DeVICE MAINTENENCE AND DISPOSAL

The AnapnoGuard endotracheal tube ETT and the connection kit are sterile, singe use components, which need to be properly disposed after extubation.

The AnapnoGuard 100 system should be disinfected and cleaned after each patient according to Hospitech Respiration LTD instructions.

# 8. Statistical Consideration

## 8.1 Safety Analysis Set

The safety analysis set will consist of all subjects for whom the study treatment was initiated. AE's will be tabulated by treatment arm, severity and relation to treatment.

## 8.2 Sample Size considerations

Presentation of sample size is based on achieving 80% power overall to demonstrate that the *rate of AE and SAE as a result of using AnapnoGuard 100 during mechanical intubation* is up to 0.15. Thus, the determination of sample size in this trial will be based on *rate of AE and SAE as a result of using AnapnoGuard 100 during mechanical intubation*.

A former study using *AnapnoGuard 100* during mechanical intubation revealed no related AE or SAE during intubation (all together 29 patients were studied). Consequently, we feel that 80% (of study patients with no AE or SAE) to be an appropriate clinical target for AnapnoGuard 100 success rate. Assuming a true success rate of 80%, a sample of **N = 25** will provide 80% power to show that AnapnoGuard 100 success (no AE or SAE during intubation) is at least 80%. This is based on a power analysis using the normal approximation for the binomial, with one-sided Alpha=0.05.

As control treatment is assumed to have the same success rate (at least 80% cases with no AE or SAE during intubation), therefore same number of patients will be recruited to the control group **(N=25).**

## To account for an approximate 15% dropout rate, we set a total number of patients N=60 (for study and control groups together).

## 8.3 Statistical Analysis

Safety of the AnapnoGuard 100 system will be evaluated by rate of AE or SAE during intubation in ICU. It is assumed that no AE or SAE will be detected wile using AnapnoGuard 100 during intubation in ICU in at least 80% of study patients.

Study hypothesis is stated in terms of Overall Proportion of Success (no AE or SAE), which is computed by total number of successes on the parameter divided by the total number in its respective cohort.

$$\text{P(success) }=\text{ }\frac{\text{Total \# of Successes}}{\text{Total \# in Cohort}}$$

The following hypothesis is specified and will be tested:

The safety proportion of success (No AE or SAE while using AnapnoGuard 100 during intubation -π_R_):

H_0_: π_R_ < 0.80

H_1_: π_R_ > 0.80

This hypothesis will be tested by:

1. Constructing a one-sided, lower 95% confidence interval about the observed Overall Proportion of Success in the relevant cohort
2. Examining whether the lower limit of the confidence interval is at or above the success criterion

Study success will be declared if the following is met:

1. Lower confidence limit of *rate of AE and SAE as a result of using AnapnoGuard 100 during mechanical intubation* success is at 0.80 or above.

## 8.4 Statistical Methods

Descriptive statistics for all parameters will be calculated. For continuous parameters descriptive statistics including mean, standard error, median, minimum, and maximum values will be reported overall and at each time-point. For ordinal parameters, counts and percentages may be reported in addition to the mean, standard error, median, minimum, and maximum values. Categorical parameters will have counts and percentages reported overall and at each time-point. Line graphs and bar charts may be displayed when a detailed description of the data necessitates.

# 9. ADVERSE EVENTS CONSIDERATION

## 9.1 Definitions Used in This Study to Determine Severity of Adverse Event (AE)

- Mild – Event that is noticeable but transient, requires no treatment, and does not interfere with the subject’s daily activities.
- Moderate – Sign or symptom which may be ameliorated by simple therapeutic measures; may interfere with usual activity.
- Severe - Signs or symptoms that are intense or debilitating and that interfere with usual activities. Recovery is usually aided by therapeutic measures and the discontinuation of the study device may be required.

## 9.2 Definition of Serious Adverse Event (SAE)

Any adverse event experience that results in any of the following outcomes:

- Death
- A life threatening event
- Requires or prolongs inpatient hospitalization or re hospitalization
- Persistent or significant disability/incapacity
- Medical or surgical intervention to avoid impairment of body function

## 9.3 Guidelines to Determine the Relationship of an AE/SAE to the Study Device/Procedure

- Definite: The adverse event is clearly related to the investigational treatment.
- Probable: The adverse event is likely related to the investigational treatment.
- Possible: The adverse event may be related to the investigational treatment.
- Unlikely: The adverse event is doubtfully related to the investigation treatment.
- Unrelated: The adverse event is clearly NOT related to the investigational agent(s).

Potential Adverse Events that may occur during the study in both the study (AnapnoGuard) and control groups are related to hyper/hypo inflation of the ETT cuff and suction dysfunction. The Adverse Events are listed in the table below:

| **Potential Adverse Events** | | **Severity** |
| --- | --- | --- |
| Over inflation of the cuff resulting in: | Hoarseness / Throat pain | Mild |
|  | Tracheal Stenosis | Moderate - Severe |
|  | Tracheal rupture | Severe |
| Under inflation of the cuff resulting in: | Leakage detected by ventilator | Mild |
|  | Decrease in saturation | Mild - Moderate |
|  | Apnea | Severe |
| Suction Dysfunction resulting in: | Tracheal Edema | Mild |
|  | Mucosal Injury | Mild - Moderate |
|  | Tracheal Ulcerations | Moderate |

## 9.4 AE Regulatory Reporting Procedures

- Any clinical study event that is judged to be an AE should be recorded on the CRF and on the AE form during the course of the study. The PI and/or study coordinator ensures this information is captured during every study subject visit. This form and the information remain a part of the CRF and are filed appropriately. All supportive information is filed in the source document.
- Whenever a study subject has reported **any** AE, the study coordinator discusses the event immediately (within **48 hours** after learning of the event) with the PI (if possible while the study subject is there). The medical independent committee must evaluate the event.
- If the AE is not serious, the information is recorded on the CRF, managed medically as appropriate, and the event is followed until resolution.
- Any SAE (according to the definitions above) will be reported by the PI to the sponsor’s representative immediately (within **24 hours** after learning of the event) for accurate and timely recording and reporting. The SAE is also recorded on the CRF, and on the SAE Form, managed medically as appropriate and the event is followed until resolution. Any relevant information available on the event (hospital records, lab tests, discharge summaries, etc.) will be applied to determine whether the SAE is related to the investigational procedure and requires reporting the event to the relevant Health Authority and/or local health authorities. Any additional information on the SAE should be also forwarded to the sponsor’s representative for further follow-up.
- **All SAE reports shall be sent to all IRB’s within 7 days (or according to local Health Authority instructions) of learning of event occurrence**.
- In addition, the PI reviews all completed AE forms for determination of SAE that require reports to Hospitech Respiration Ltd.
- For reasons of confidentiality, subject identifying data (e.g., name, social security numbers) will not be used; instead, a study-specific code number should be used.

Hospitech Respiration Ltd representatives will provide direction for consistent and systematic handling of such reports and determine their reportability to the local Health Authority. Only related unexpected SAE’s will be reported to the Health Authority. Therefore, not all SAEs/Product Complaints reported to Hospitech Respiration Ltd will require subsequent notification to the Health Authority. However all study investigators will be informed by clinical study personnel of any Serious Adverse Events (SAE) and product complaint reported to the Health Authority associated/related to the medical device used in this study.

## 9.5 Adverse Events Reported by the Sponsor (IND Safety Reports)

All investigational new device (IND) safety reports (AE/SAE) received from Hospitech Respiration Ltd will be forwarded to the PI. The PI will send copies to all staff involved in the study.

The PI and/or study coordinator will file copies of these SAE and AE in the regulatory binder for that study, also known as the site file.

## 9.6 List of Serious Adverse Events

Serious, procedure related adverse events include those events with one or more of the following outcomes:

- Death: The subject’s death is suspected as being a direct outcome of the adverse event.
- Life-Threatening: The subject was at eminent risk of dying at the time of the adverse event or it is suspected that the use of the device or continuation of the procedure would have resulted in the subject’s death.
- Hospitalization (initial or prolonged): The use of the device or procedure was associated with an adverse event that resulted in the subject’s readmission to the hospital (>24 hours) or the prolongation of a hospital stay by >24 hours beyond the standard of care in the specific site.
- Disability: The use of the device or procedure was associated with an adverse event that resulted in significant, persistent, or permanent change, impairment, damage, or disruption in a subject’s body function/structure, physical activities, or quality of life.
- Requires Intervention to Prevent Permanent Impairment or Damage of a Body Part and/or Function: The use of the device or procedure resulted in a condition that required medical or surgical intervention to preclude permanent impairment or damage to a subject.

## 9.7 Clinical Management of Study Related Adverse Events

AEs will be clinically managed according to medical indications, Local Health Authority, GCP and the standard of care in the specific Medical Center. Subjects will be entitled for continuation of clinical follow-up and treatment until of their study related clinical problem has been resolved, even beyond the pre-defined 1 month follow-up period.

## 9.8 Product Complaints

The company conducted extensive Lab and animal experiments as well as a successful pilot clinical trial in order to establish the safety of the system with regards to procedural safety and biodegradability. All these study results are present in the Investigator Brochure. Based on all data and reports, the Company has concluded that the product is safe to be used in human subjects. However, all product complaints on devices cleared for distribution are to be evaluated by the manufacturer to determine whether the complaint represents an event, which is required to be reported to local Health Authority, under Medical Device Reporting. To comply with this regulation, product complaints are defined as: “Concerns about deficiencies related to the identity, quality, durability, reliability, safety, effectiveness, or performance of the device.”

## 9.9 Criteria for subject’s Study Termination

Termination of the study may be due to any of the following reasons:

- Decision by the investigator or sponsor that termination is in the subjects’ best medical interest.
- Device failure.
- Other ethical or clinical considerations.

# 10. Study Monitoring

## 10.1 Study Records/Source Document Inspection

The investigator will allow representatives of Hospitech Respiration Ltd, its monitoring team, the Health Authority or other governmental regulatory agencies to audit all study records, Worksheets, IRB/Ethical Committee records, and corresponding portions of the subject’s office and/or hospital medical records at regular intervals throughout the study. These inspections are conducted to verify adherence to the protocol, integrity of the data being captured on the Worksheets and compliance with applicable regulations. Hospitech Respiration Ltd agrees that subjects’ medical records will be maintained in a confidential manner. Study reports will not identify subjects by name.

## 10.2 Monitors

The study will be monitored by a representative of Hospitech Respiration Ltd at regular intervals throughout the course of the study according to a pre-defined monitoring plan. On site monitoring of the investigator’s facilities aids will be conducted in order to ensure compliance with the protocol.

Any deficiency noted during the monitoring visits will be discussed with the investigator and the corrective actions to be taken will be agreed upon. Should the sponsor determine, at any time during the study that the investigator does not comply with the regulatory /protocol compliance, measures necessary to establish compliance will be implemented. If compliance cannot be maintained, the sponsor will suspend or terminate the study at this site.

## 10.3 Monitoring Plan

**I. Pre-study visit**

1. Assess the site’s infrastructure (staff and facility) for the capability to conduct the study.
2. Evaluate Investigator and staff’s Experience & Qualifications
3. Financial Disclosure information.
4. Hospital Laboratory.

**II. Study Initiation visit**

Orients the investigator’s staff involved in the study on:

1. The device, its intended use and how to operate it;
2. The study protocol;
3. CRF;
4. GCP and other regulatory requirements;
5. Informed Consent form and process;
6. AE, SAE, Safety reporting;
7. IRB/Ethical Committee;
8. Subject information- subject’s identification log, subject screening log, subject enrollment log, subject study visit log;
9. Study monitoring;
10. Investigator’s study files;
11. Report of AE back from the Sponsor to the site;
12. Expectations from the site regarding data collection and timelines.

**III. Regular Monitoring visits**

1. Check on the progress of the study;
2. Protocol and GCP compliance;
3. Informed Consent form and process;
4. CRF completion, correction, source data verification;
5. AE, SAE, Safety reporting;
6. Investigational product maintenance;
7. Investigator’s study files.

Detailed monitoring schedule to be provided at study initiation

**IV. Final Study visit - Close out monitoring visit**

Ensure Investigator understands the on-going responsibilities

1. Record archival practices for source documents & CRF’s after completion of the study - up to 15 years;
2. Follow-up of on-going Adverse Events - up to 30 days after completion of the study;
3. Notify the sponsor in the event of Health Authority inspection.

# 11. Confidentiality/Publication of Study Results

This clinical study is confidential and should not be discussed with individuals outside the study. Additionally, the information in this document and in the study may contain secrets and commercially sensitive information that is confidential and may not be disclosed unless such disclosure is required by federal or state law or regulations. Subject to the foregoing, this information may be disclosed only to those persons involved in the study that have a need to know, but all such persons must be instructed not to further disseminate this information to others.

The data may be used now and in the future for presentation or publication at the investigator and/or sponsor’s discretion or for submission to governmental regulatory agencies.

All reports and communications relating to subjects in the study will identify each subject only by the subject’s code (subject’s initials and subject’s study number).

# 12. Record Keeping

## 12.1 Source Documents

Source documents are the initial documents whereon subject data are recorded. This includes, but is not limited to, original subject files, hospital records, and original recordings/tracings from automated instruments (e.g., ECG, Blood tests, etc.), X-ray films, and laboratory notes. The information contained within the source documents is used to complete the CRF worksheets.

All information captured on the CRF worksheets should be accurately supported by the source documents unless specifically approved by Hospitech Respiration Ltd and written so in a certification document filed in the site and study folders. (e.g., Subject questionnaires will be cleared to be included as part of the CRF worksheets and considered as source documents).

For example, each subject’s source documents should include (but not be limited to):

- After subject was enrolled to the study mention the date of subject's signature on Informed Consent and the name of the protocol
- Subject full name and identification
- Date of each study required visit with a description of the visit and the results of each procedure that was performed
- All concomitant procedures
- VAS (Visual Analogue Scale) Scale;

Any additional information relevant to the study should be included in the subject’s source documents. In particular, any deviations from the study protocol or procedures should be recorded in the source documents. For example, if study required procedures or visits are not completed or are completed outside the time frame specified in the protocol, the reasons for the departure should be explained in the source documents. The investigator must maintain all study documentation at least 2 years after the last approval of marketing application in and until there are no pending or contemplated marketing application or at least 2 years have elapsed since the formal discontinuation of clinical development of the investigational product. These documents should be retained for a longer period, however, if required by the applicable regulatory requirements or by an agreement with the sponsor. The sponsor should inform the investigator in writing when the trial-related records are no longer needed.

## 12.2 Data Collection Method

Data from the subject’s permanent medical records (see source documentation section) will be recorded on worksheets supplied by the investigator and approved by the sponsor. These worksheets (comprising the CRF's) will be used to transmit the information collected in the performance of this study to the clinical database either manually or by site data entry (a third party vendor may be used for this).

CRF’s should be filled no longer than 2 weeks after each visit.

All worksheets must be typewritten or filled out in black ink, accurately and promptly following each examination or surgery. No questions will be left blank. Corrections will be made only by lining out (single line) incorrect data and writing in the revisions. All corrections must be initialed and dated by the individual performing/recording the correction. If the reason for the change is not obvious, an explanation for the change will be recorded. Blacking out or using correction fluid or an eraser will not be used to eliminate data. The investigator must review the worksheets for completeness and accuracy and must sign/date the forms where indicated. Signature stamps or substitutes are not acceptable. The investigator will retain originals of all source documents, subject consent forms, and study data as a permanent record.

Each set of original worksheets should be reviewed for accuracy and completion (signatures, dates, adverse events, serious adverse events, protocol departures) and maintained in the investigator’s study files.

# 13. REGULATORY OBLIGATIONS

## 13.1 Sponsor's Obligations

Clinical research studies are subject to the local Institutional Review Board committee’s approval. A Sponsor must assume the following responsibilities and must keep the required records. The Sponsor must:

1. Provide the Investigator with the necessary information- Protocol and Device User's Manual or Instructions for Use.
2. Inform the Investigator of all new information that may affect his/her decision of whether to continue their participation in the study.
3. Provide the supplies (AnapnoGuard system and disposable parts) for the investigation.
4. Provide Clinical Study Insurance Policy

Maintain the following records:

1. A signed protocol and CRF
2. All correspondence that relate to the clinical trial
3. Signed Investigator Agreement
4. Records of device shipments: For systems (Dates, Qty and S/Ns) for disposable tubes and kits (Dates, Qty and lot # ). Systems will be shipped back to the sponsor at the end of the study and the disposable tubes will be disposed by the hospital staff according to the ICU internal procedure.
5. Other records as required by the Health Authority.

## Investigator's Obligations

Clinical research studies are subject to the regulations of the local Health Authority. Upon signing the protocol, the Investigator agrees to assume the following responsibilities and to keep the required records for a period of three years following completion of the study and to file the required reports in a timely manner:

1. Conduct the Investigation in compliance with the protocol. Changes to the protocol may only be made after approval by the Sponsor, or when necessary to protect the safety, rights or welfare of a subject.
2. Personally conduct or supervise the investigation.
3. Read and understand the information in the Protocol and Investigator Brochure.
4. Be aware of the potential risks and side effects of implanting the AnapnoGuard system.
5. Ensure that all associates, colleagues and employees assisting in the conduct of the study are qualified, properly trained on the use of the device and are informed about their responsibilities and obligations related to the study.
6. Inform all subjects that the device is being used for investigational purposes and ensure that the requirements related to obtaining the informed Consent are met.
7. Screen patients and enroll to the study only patients who meet the inclusion criteria.
8. Dispose of remaining supplies as directed by the Sponsor.

Maintain the following records (for a period of three years following completion of the study):

1. Signed copy of the protocol.
2. Signed Inform Consent forms.
3. All correspondence as relates to the clinical trial.
4. Case Report Forms.

File the following reports:

1. Severe Adverse Events must be reported to the sponsor by phone within 24 hours. A written report must follow within five (5) working days.
2. Unanticipated adverse device related effects must be reported to the sponsor as soon as possible, within 10 working days.
3. Deviations, which were made from the protocol for emergency use, must be reported to the sponsor as soon as possible, but no later than five (5) working days after its occurrence.

# Study Completion

The PI will complete and report the study in satisfactory compliance with the protocol.

It is agreed that for any reasonable cause, either the PI or the sponsor, Hospitech Respiration Ltd, may terminate this study, provided a written notice is submitted at a reasonable time in advance of intended termination. If the study is terminated for safety reasons, the investigator will be notified immediately by telephone, followed by written instructions for study termination notification of the IRB/Ethical Committee.

# Protocol Deviations and Exceptions

The investigator should not implement any deviation from- or changes of the protocol without written agreement in advance of the agreement by the sponsor and prior review and documented approval from the local Institutional Review Board committees of an amendment, except where necessary to eliminate an immediate hazard(s) to trial subjects.

The investigator should document and explain any deviation from the approved protocol. The reasons for it, and, if appropriate, the proposed protocol amendments should be submitted:

1. To the Sponsor for agreement
2. To the local IRB/Ethical Committee
3. To the local regulatory authority – if needed.

# Removal of Subjects from the Study

A subject has the right to withdraw from the study at any time, for any reason, without prejudice to her future medical care by the physician or the institution. Should a subject (or the subject’s legally acceptable representative) decide to withdraw, all efforts will be made to collect and report the final visit observations as thoroughly and timely as possible.

Subjects may be removed from the study if any one or more of the following events occur:

- Refusal of the subject to continue treatment and/or observations;
- Decision by the Investigator that termination is in the subject’s best medical interest;
- Subject is lost to follow-up;
- Other ethical or clinical considerations.

# 17. Ethical Conduct of the Study

This study will be conducted in compliance with the protocol after approved of the local Institutional Review Board committees, and according to Good Clinical Practice (GCP) standards.

No deviation from the protocol will be implemented without the prior review and approval of the Health Authorities except where it may be necessary to eliminate an immediate hazard to a research subject. In such case, the deviation will be reported to the Health Authorities as soon as possible.

A copy of the protocol, Informed Consent Form (ICF) and advertising material must be submitted to the Health Authorities. Written approval of the protocol and Informed Consent Form must be obtained prior to subject enrollment.

**18.** **References**

1. Stauffer JL, Olson DE, Petty TL. Complications and consequences of endotracheal intubation and tracheotomy: a prospective study of 150 critically ill adult patients. Am J Med 1981;70: 65–76.
2. Lewis FR Jr, Schiobohm RM, Thomas AN. Prevention of complications from prolonged tracheal intubation. Am J Surg 1978; 135:452–7.
3. Klainer AS, Turndorf H, Wu WH, et al. Surface alterations due to endotracheal intubation. Am J Med 1975;58:674–83.
4. Ferdinande P, Kim DO. Prevention of postintubation laryngotracheal stenosis. Acta Otorhinolaryngol Belg 1995;49:341–6.
5. Ferrer M, Torres A. Maintenance of tracheal tube cuff pressure: where are the limits? Crit Care. 2008;12:106- -7.
6. Scheld WM. Developments in the pathogenesis, diagnosis and treatment of nosocomial pneumonia. Surg Gynecol Obstet 1991; 172:42–53.
7. Garrouste-Orgeas M, Chevret S, Arlet G, et al. Oropharyngeal or gastric colonization and nosocomial pneumonia in adult intensive care unit patients. Am J Respir Crit Care Med 1997;156: 1647–55.
8. Guidelines for prevention of nosocomial pneumonia. Centers for Disease Control and Prevention. Respir Care 1994;39: 1191–236.
9. Spray SB, Zuidema GD, Cameron JL. Aspiration pneumonia: incidence of aspiration with endotracheal tubes. Am J Surg 1976;131:701–3.
10. Craven DE, Steger KA. Epidemiology of nosocomial pneumonia: new perspectives on an old disease. Chest 1995;108: 1–16.
11. American Thoracic Society; Infectious Diseases Society of America. Guidelines for the management of adults with hospital-acquired, ventilator-associated, and healthcare-associated pneumonia. Am J Respir Crit Care Med. 2005;171: 388-416.
12. Rello J, Soñora R, Jubert P, Artigas A, Rué M, Vallés J. Pneumonia in intubated patients: role of respiratory airway care. Am J Respir Crit Care Med. 1996; 154:111-5.
13. Valencia M; Ferrer M; Farre R; Navajas D; Badia JR; Nicolas JM; Torres A. Automatic control of tracheal tube cuff pressure in ventilated patients in semirecumbent position: a randomized trial. Crit Care Med. 2007 Jun;35(6):1543-9.
14. Nseir S, Duguet A, Copin MC, De Jonckheere J, Zhang M, Similowski T, Marquette CH. Continuous control of endotracheal cuff pressure and tracheal wall damage: a randomized controlled animal study. Crit Care. 2007;11(5):R109.
15. Efrati S, Leonov Y, Oron A, Siman-Tov Y, Averbukh M, Lavrushevich A, Golik A. Optimization of endotracheal tube cuff filling by continuous upper airway carbon dioxide monitoring. Anesth Analg. 2005; 101:1081-8.
